# Biodiversity and natural capital in ecologically sensitive regions

**DOI:** 10.1038/s41598-026-63956-4

**Published:** 2026-07-27

**Authors:** Vishal Chettry, Rakshak Kumar, Riccardo Testa

**Affiliations:** 1 Department of Architecture, Planning & Design, IIT(BHU), Varanasi, Uttar Pradesh 221005 India; 2 Department of Molecular Biology and Bioinformatics, Tripura University, Suryamaninagar, Agartala, Tripura India; 3 Department of Agricultural, Food and Forest Sciences, University of Palermo, Palermo, Italy

**Keywords:** Biodiversity, Natural capital, Ecosystem, Ecology, Habitat, Biodiversity, Ecosystem ecology

## Abstract

Biodiversity and natural capital support economic systems, human well-being and climate resilience. Yet conservation and planning have focused mainly on visible ecosystems such as forests, wetlands and agricultural landscapes, while overlooking belowground biodiversity and the complex interactions between rural and rapidly urbanizing regions. This Collection analyses species, community and microbiome responses to environmental gradients, management interventions and climate constraints, and the effects of these responses on productivity, habitat quality, ecosystem functioning and natural capital. Several contributions highlight that climate-adapted seed sourcing in grand fir can maintain forest growth and carbon sequestration under changing moisture regimes, and that diverse multi-crops in boreal conditions raise biomass and net energy yields while lowering environmental pressures. Other studies introduce integrated indicators such as habitat quality indices for wetland waterfowl, soil functional networks in shaded coffee systems centred on total organic carbon, and multidimensional niche assessments for zooplankton communities. Together, these papers demonstrate complementary approaches for treating biodiversity as natural capital and for sustaining the ecosystem services that support human well-being, sustainable production and informed conservation and management decisions.

## Introduction

Biodiversity conservation and natural capital restoration have traditionally focused on visible components of ecosystems such as agricultural lands, forests, grasslands, wetlands, wildlife and freshwater. Yet an equally important layer of biodiversity lies belowground in the form of soil microbial communities, which regulate nutrient cycling, organic matter turnover, carbon storage, soil formation, water retention and plant productivity, and thereby underpin ecosystem health and resilience^1^. In fragile hilly and mountainous landscapes, where steep gradients, thin soils and rapid environmental change intensify vulnerability, these hidden microbial processes are especially critical for stabilising slopes, supporting vegetation and aiding recovery after disturbances such as landslides or glacier retreat^2^. As restoration efforts expand in ecologically sensitive regions, there is a growing need to move beyond a purely aboveground focus and integrate soil microbiota and other belowground processes into conservation and planning, so that both visible biodiversity and the less visible foundations of natural capital are restored together^3^.

Moreover, biodiversity and natural capital are under growing pressure as rapid urban expansion transforms ecological systems, particularly in ecologically sensitive hilly and mountainous regions^4^. From an ecological planning perspective, cities often sprawl into forested slopes, river valleys and fragile ridgelines, fragmenting habitats and altering hydrological cycles that underpin services such as clean water, soil stability and local climate regulation^5^. The loss of natural capital through deforestation for housing, road building, tourism infrastructure and quarrying reduces the capacity of these landscapes to buffer disasters such as landslides, floods and heatwaves, even as demand for ecosystem services rises. Integrating biodiversity and natural capital into land-use plans, zoning and infrastructure design is therefore critical, including protecting native vegetation, securing riparian buffers, connecting ecological networks and valuing services such as carbon storage and pollination^6^. In hilly, mountainous and other ecologically sensitive areas, conservation-oriented urban design and strict regulation of land conversion are essential to sustain both nature’s intrinsic value and the economic and social benefits that resilient ecosystems provide to growing urban populations^7^.

Ultimately, natural capital provides significant economic benefits through ecosystem services such as food production, water purification, carbon sequestration, climate regulation, renewable energy production, soil fertility, pollination and disaster risk reduction^8^. Furthermore, the maintenance and development of biodiversity, on the one hand, allows for diversification and differentiation of the production portfolio, whilst, on the other, it ensures greater resilience of various ecosystems as well as to climate change, thereby guaranteeing higher economic benefits^9^. Therefore, ecosystem degradation and biodiversity loss represent not only an environmental concern, but also a significant social and economic risk. Recognition of these intrinsic and extrinsic dimensions of value underscores the importance of sustainable ecosystem management approaches for long-term planning, through which present and future generations can benefit from sustainable economic development^10^.

Overall, the Biodiversity and Natural Capital Collection at *Scientific Reports* brings together research that spans organisms, ecosystems and the services they provide, from forests and agroecosystems to aquatic communities and the ocean twilight zone. Across these contributions, authors examine species and community responses to environmental gradients, management practices and climate constraints^11^, and the effects of these responses on productivity, habitat quality and ecosystem functions^12,13^. The Collection also highlights the importance of functional indicators, sustainable productions, microbiomes and traditional ecological knowledge^14,15^ for understanding and managing nature as a form of natural capital. Together, these studies point to diverse but complementary ways of sustaining biodiversity and the ecosystem services that support human well-being.
